# Nosocomial Pneumonia in Georgia: A Focus on Gram-Positive Bacteria and Antimicrobial Resistance

**DOI:** 10.7759/cureus.77710

**Published:** 2025-01-20

**Authors:** Giorgi Mgeladze, Giorgi Akhvlediani, Shorena Khetsuriani, Giorgi Maisuradze, Vakhtang Robakidze, Shota Mrelashvili, Ani Papiashvili

**Affiliations:** 1 Microbiology, Georgian American University, Tbilisi, GEO; 2 Pulmonary and Critical Care Medicine, Tbilisi State Medical University, Tbilisi, GEO; 3 Biomedical Sciences, Georgian American University, Tbilisi, GEO; 4 Microbiology, Tbilisi State Medical University, Tbilisi, GEO

**Keywords:** georgia, gram-positive bacteria, meca gene, methicillin-resistant staphylococcus aureus (mrsa), multi-drug resistant bacteria, nosocomial pneumonia, pbp 2

## Abstract

Nosocomial pneumonia represents a significant clinical challenge worldwide, and in Georgia, the burden of this healthcare-associated infection is a growing concern. This study investigates the role of gram-positive bacteria in nosocomial pneumonia cases, focusing on their prevalence, antimicrobial resistance patterns, and associated risk factors. A retrospective analysis of 484 clinical samples collected from 397 patients between May 2022 and September 2024 highlights the distribution of pathogens, with a particular emphasis on *Staphylococcus aureus* and *Streptococcus pneumoniae*. Among gram-positive pathogens, *Staphylococcus aureus* was the most prevalent, accounting for 103 cases (21.3%), followed by *Streptococcus pneumoniae* with 45 cases (9.3%).

The study identifies alarming rates of antimicrobial resistance among gram-positive pathogens. *Staphylococcus aureus* isolates demonstrated universal penicillinase production (103/103, 100%) and high levels of mecA-mediated methicillin resistance (89/103, 86.4%) and erm-mediated macrolide resistance (74/103, 71.8%). Additionally, notable resistance was observed to tetracycline (93/103, 90.3%), aminoglycosides (31/103, 30.1%), and fluoroquinolones (41/103, 39.8%). *Streptococcus pneumoniae* isolates exhibited universal penicillinase production (45/45, 100%), with complete beta-lactam resistance found in 42 isolates (42/45, 93.3%), mediated through mutations in the pbp1a, pbp2x, and pbp2b genes. Furthermore, erm(B)-mediated macrolide resistance was observed in 37 isolates (37/45, 82.2%), tetM-mediated tetracycline resistance in 37 isolates (37/45, 82.2%), and fluoroquinolone resistance in 13 isolates (13/45, 28.9%). One isolate of each pathogen demonstrated vancomycin resistance, underscoring the emergence of multidrug-resistant (MDR) strains.

The study underscores the need for stringent infection control measures and rational antibiotic stewardship to mitigate the impact of resistant gram-positive pathogens in Georgian healthcare settings. The findings also stress the importance of continuous surveillance to monitor resistance trends and guide empirical therapy. By exploring the resistance mechanisms and prevalence of gram-positive bacteria in nosocomial pneumonia, this research contributes to a deeper understanding of the local epidemiology and highlights actionable insights for improving patient outcomes.

## Introduction

Nosocomial pneumonia, one of the most common healthcare-associated infections, remains a critical concern for medical institutions worldwide. Defined as pneumonia acquired 48 hours or more after hospital admission and not present at the time of admission, this condition is one of the leading causes of morbidity and mortality in hospitalized patients, as evidenced by global studies [1,2]. In Georgia, like in many other countries, the burden of nosocomial pneumonia continues to rise, reflecting challenges in infection control, antimicrobial stewardship, and the growing prevalence of resistant pathogens [3].

Gram-positive bacteria, particularly *Staphylococcus aureus* and *Streptococcus pneumoniae*, are primary causative agents of nosocomial pneumonia [4]. These pathogens are associated with significant clinical and economic impacts due to their ability to develop resistance to multiple antibiotics [5]. Infections caused by *Staphylococcus aureus* are frequently complicated by methicillin resistance mediated by the mecA gene [6], while *Streptococcus pneumoniae* isolates often harbor mutations in penicillin-binding proteins (pbp1a, pbp2x, and pbp2b) [7] or erm(B)-mediated macrolide resistance, contributing to treatment failures and prolonged hospital stays [8].

In Georgian healthcare settings, there is a growing recognition of the critical role antimicrobial resistance plays in nosocomial infections. However, comprehensive data on the prevalence and resistance profiles of gram-positive pathogens in nosocomial pneumonia are scarce. This lack of data impedes the development of targeted treatment guidelines and hampers infection control efforts. The emergence of resistance to multiple antibiotic classes, including beta-lactams, macrolides, tetracyclines, and aminoglycosides, further exacerbates the problem, leaving clinicians with fewer effective therapeutic options [9].

This study critically examines the prevalence and resistance patterns of gram-positive bacteria in nosocomial pneumonia within Georgia. By retrospectively analyzing data from clinical samples collected over two years, we aim to provide an in-depth understanding of the distribution of these pathogens and the genetic mechanisms underlying their resistance. In doing so, this research contributes to the development of evidence-based strategies to combat antimicrobial resistance, improve patient outcomes, and inform local and global efforts in tackling healthcare-associated infections [10].

The findings of this study are expected to serve as a foundation for strengthening infection prevention and control practices, optimizing empirical treatment protocols, and fostering antibiotic stewardship initiatives in Georgia. By focusing on gram-positive bacteria in nosocomial pneumonia, this research provides critical insights into an area of increasing clinical and public health importance, with implications for both local healthcare systems and the broader global health context [10].

## Materials and methods

Study design and sample collection

This retrospective study analyzed 484 clinical samples collected from May 2022 to September 2024 from patients diagnosed with nosocomial pneumonia in Georgian hospitals. Samples were obtained from three major cities in Georgia, ensuring representation across different regions and healthcare settings. Specifically, 368 samples (76.03%) were collected from university hospitals in Tbilisi, while 62 samples (12.8%) and 54 samples (11.6%) were collected from community hospitals in Kutaisi and Batumi, respectively. These specimens were derived from patients' pulmonary samples, comprising 422 sputum samples (87.2%) and 62 bronchoscopy samples (12.8%). To minimize contamination risks, patients were gargled with normal saline three times before sample collection. Lower respiratory tract secretions were then collected using either a sterile sputum collector or a fiberoptic bronchoscope and placed into sterile containers. Clinically significant values for quantitative culture were defined as 20 polymorphonuclear leukocytes per low-power field (LPF) to ensure the quality and reliability of the samples.

Inclusion Criteria

Adults aged 18 and above diagnosed with nosocomial pneumonia were included if the diagnosis was confirmed by radiological evidence (new or progressive infiltrates) and microbiological evidence (positive cultures from respiratory specimens), with onset occurring 48 hours or more after hospital admission.

Exclusion Criteria

The study excluded patients with community-acquired pneumonia, prior antibiotic treatment within 14 days, severe immunosuppression, chronic lung diseases, concurrent infections, or those under 18 years of age.

Pathogen identification

Pathogen identification was performed using two methods. MALDI-TOF MS (Matrix-Assisted Laser Desorption/Ionization-Time of Flight Mass Spectrometry) was used for rapid and precise identification of bacterial species from cultured isolates [11]. Additionally, biochemical identification was carried out using API test kits (bioMérieux), including API 20E, API NE, API Staph, and API Strep, to analyze gram-negative bacteria, staphylococci, and streptococci, respectively [12].

Antimicrobial susceptibility testing

Antimicrobial susceptibility testing was performed on freshly cultured Staphylococcus aureus and Streptococcus pneumoniae isolates using the Kirby-Bauer disk diffusion method, following European Committee on Antimicrobial Susceptibility Testing (EUCAST) guidelines [13]. Bacterial suspensions were standardized to a 0.5 McFarland turbidity, corresponding to approximately 1.5 × 10⁸ CFU/mL. These suspensions were uniformly inoculated onto Mueller-Hinton agar plates. Antibiotic-impregnated disks were placed on the agar surface, and the plates were incubated at 37°C for 18-24 hours. After incubation, inhibition zone diameters were measured and interpreted as "susceptible," "susceptible, increased exposure," or "resistant" based on the latest EUCAST breakpoint tables (version 14.0).

The antibiotics tested included imipenem (10 µg), meropenem (10 µg), ertapenem (10 µg), amikacin (30 µg), gentamicin (10 µg), levofloxacin (5 µg), cefoxitin (30 µg), oxacillin(10 µg), ampicillin (10 µg), clindamycin (2 µg), erythromycin (15 µg), linezolid (30 µg), teicoplanin (30 µg), rifampin (5 µg), and tetracycline (30 µg). Isolates exhibiting resistance to at least one agent in three or more antimicrobial classes were classified as multidrug-resistant (MDR) [13].

Resistance mechanism analysis

**Figure 1:**
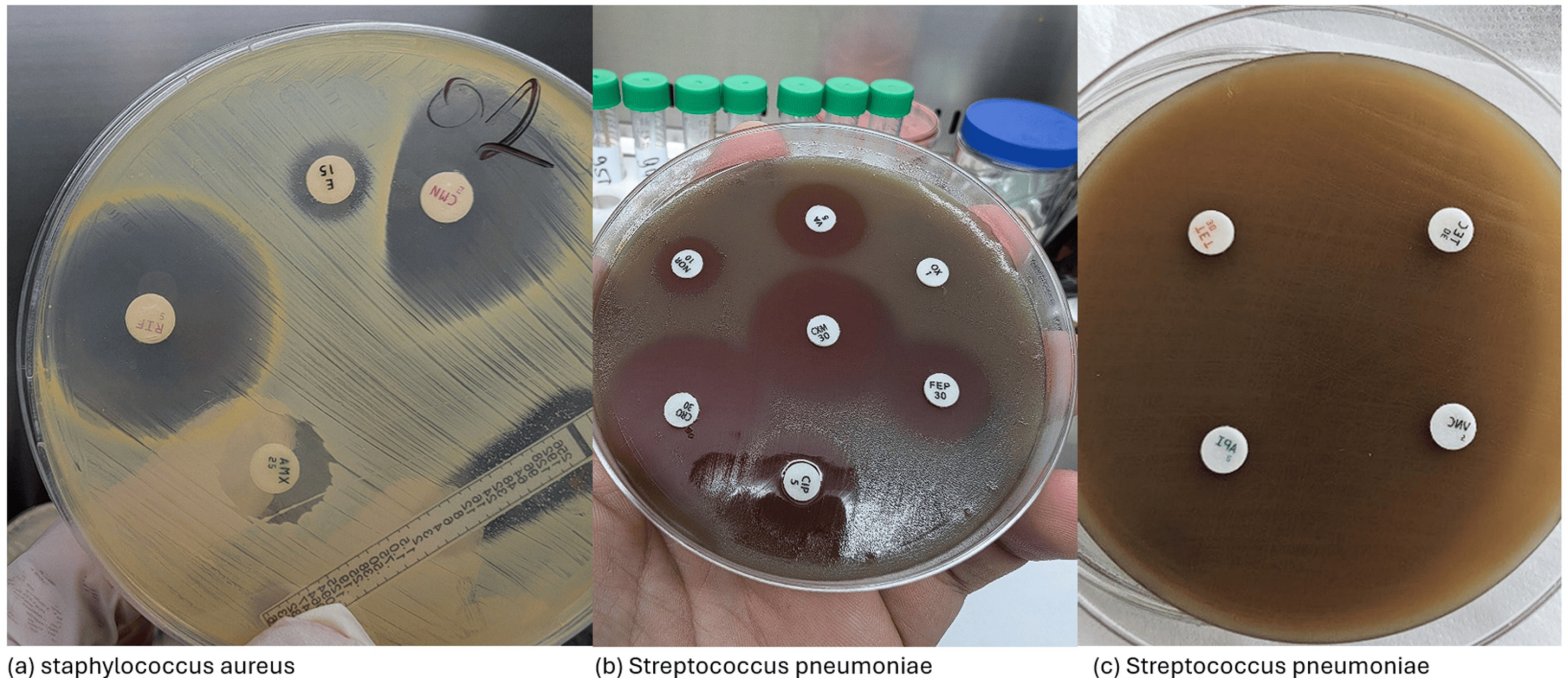
Antibiotic susceptibility testing of Staphylococcus aureus and Streptococcus pneumoniae isolates using the Kirby-Bauer disk diffusion method This figure illustrates the antibiotic susceptibility testing of *Staphylococcus aureus* (a) and *Streptococcus pneumoniae* (b, c) using the Kirby-Bauer disk diffusion method to detect resistance mechanisms. (a) The antibiotic disks used are E 15 (erythromycin, 15 µg), CMN 2 (clindamycin, 2 µg), AMX 25 (amoxicillin, 25 µg), and RIF 5 (rifampin, 5 µg). Flattening of the clindamycin inhibition zone adjacent to the erythromycin disc indicates inducible clindamycin resistance. (b) Provided as a reference for showing susceptible *Streptococcus pneumoniae*, the antibiotics tested include CIP 5 (ciprofloxacin, 5 µg), FEP 30 (cefepime, 30 µg), CXM 30 (cefuroxime, 30 µg), CRO 30 (ceftriaxone, 30 µg), VA 5 (vancomycin, 5 µg), and NOR 10 (norfloxacin, 10 µg). Clear zones of inhibition around the antibiotic disks indicate susceptibility to these antibiotics. (c) The antibiotics tested include TET 30 (tetracycline, 30 µg), API 5 (ampicillin, 5 µg), TEC 30 (teicoplanin, 30 µg), and VNC 2 (vancomycin, 2 µg). The absence of clear zones of inhibition indicates significant resistance demonstrated by *Streptococcus pneumoniae*.

By following the EUCAST guidelines, we successfully identified vancomycin-resistant Staphylococcus aureus (VMRSA) in our laboratory. Using broth microdilution, we determined that the isolates had MIC values exceeding the resistance breakpoint for vancomycin. To confirm these findings, we performed standardized disk diffusion tests, which revealed zone diameters below the established thresholds for susceptibility. For *Staphylococcus aureus*, methicillin resistance was determined using cefoxitin (30 µg) discs, and the presence of the mecA gene was used to confirm methicillin-resistant Staphylococcus aureus (MRSA). Inducible clindamycin resistance was assessed using the D-test, where erythromycin (15 µg) and clindamycin (2 µg) discs were placed on the same plate. Flattening of the clindamycin inhibition zone adjacent to the erythromycin disc indicated inducible clindamycin resistance (Figure 1a) [14]. Throughout the process, we ensured accurate inoculum preparation and strict adherence to incubation conditions to maintain result reliability. The combination of MIC determination, disk diffusion, and targeted resistance testing provided robust evidence for classifying the isolates as VMRSA [13].

For *Streptococcus pneumoniae*, resistance to penicillin and macrolides was analyzed. Genetic markers, including pbp1a, pbp2x, pbp2b, erm(B), and tetM genes, were identified to assess the underlying mechanisms of resistance (Figure 1c) [15].

As *Streptococcus pneumoniae* is always cultivated on blood agar, a clear zone of inhibition must be observed around the antibiotic disc to indicate susceptibility to the tested drug. For reference, Figure 1b clearly demonstrates inhibition zones, which are indicative of susceptibility to the antibiotics tested. Based on the Kirby-Bauer disk diffusion method, as shown in Figure 1c, the absence of a clear inhibition zone around any of the antibiotics tested indicates resistance to those antibiotics [13].

Statistical analysis

Data were analyzed using Microsoft Excel and R software (version 4.2.0) (R Core Team, 2021). Associations between pathogen types and resistance patterns were evaluated using the chi-square test and Fisher’s exact test, with a p-value of <0.05 considered statistically significant.

Ethical approval

This study was conducted in compliance with ethical standards and was approved by the Ethics Committee of Tbilisi State Medical University (#425486). All patients provided informed consent for the use of their clinical data.

## Results

**Figure 2:**
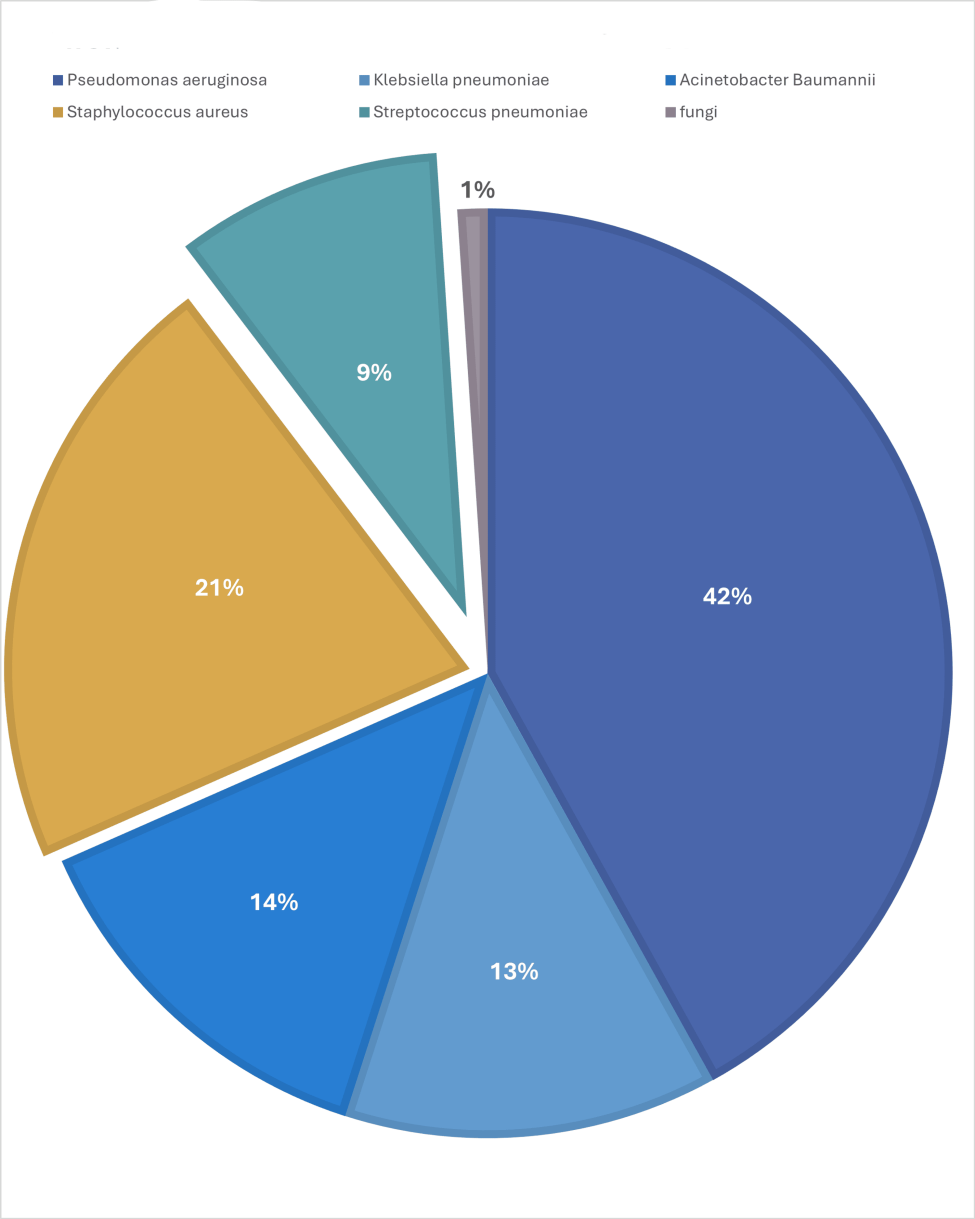
Distribution of pathogens identified from 484 clinical samples (2022 May-2024 September) This figure illustrates the distribution of pathogens identified in 484 clinical samples collected between May 2022 and September 2024. The most frequently isolated pathogen was *Pseudomonas aeruginosa* (203 isolates, 41.94%), followed by *Staphylococcus aureus* (103 isolates, 21.3%), *Acinetobacter baumannii* (65 isolates, 13.4%), *Klebsiella pneumonia* (63 isolates, 13%), *Streptococcus pneumoniae* (45 isolates, 9.3%), and fungi (5 isolates, 1%). The percentages represent the proportion of each pathogen relative to the total sample size.

In total, 484 clinical samples were collected from 397 patients over a two-year period from May 2020 to September 2024. The analysis revealed that *Pseudomonas aeruginosa* was the most frequently identified pathogen, accounting for 41.94% (203/484) of the cases. Among gram-positive bacteria, *Staphylococcus aureus* was the most common, comprising 21.3% (103/484) of all isolates, followed by *Streptococcus pneumoniae*, which accounted for 9.3% (45/484). Other pathogens included *Acinetobacter baumannii* (13.4%, 65/484) and *Klebsiella pneumoniae* (13%, 63/484). Fungal infections were rare, representing only 1% (5/484) of the total samples (Figure 2).

**Figure 3:**
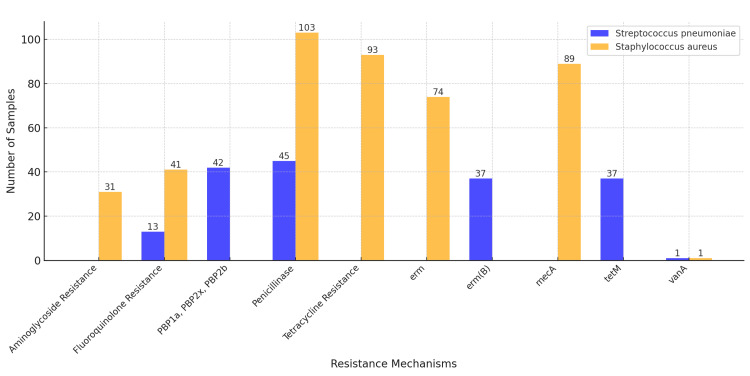
Comparison of antibiotic resistance mechanisms in Streptococcus pneumoniae and Staphylococcus aureus The figure illustrates the prevalence of antibiotic resistance mechanisms in *Streptococcus pneumoniae* and *Staphylococcus aureus*. Among the analyzed mechanisms, *Staphylococcus aureus* exhibited significantly higher rates of penicillinase production (103 samples), tetracycline resistance (93 samples), and the presence of the mecA gene (89 samples), indicating widespread resistance to β-lactam antibiotics and methicillin. In contrast, *Streptococcus pneumoniae* showed notable resistance through alterations in penicillin-binding proteins (42 samples) and the tetM gene (37 samples), with fewer cases of aminoglycoside resistance (13 samples). The vanA gene, associated with vancomycin resistance, was rare in both species, with only one sample each. This comparison underscores the differences in resistance profiles between the two pathogens, emphasizing the importance of targeted treatment strategies.

Resistance patterns among *Staphylococcus aureus* isolates demonstrated significant challenges to treatment options. All 103 isolates were found to produce penicillinase, indicating a universal resistance to penicillin. Moreover, 86.4% (89/103) of the isolates were methicillin-resistant, mediated by the mecA gene. Resistance to tetracyclines was noted in 90.3% (93/103) of isolates, while aminoglycoside resistance was detected in 30.1% (31/103). Fluoroquinolone resistance was observed in 39.8% (41/103) of the isolates. Alarmingly, one isolate exhibited resistance to vancomycin, signaling the emergence of MDR *Staphylococcus aureus* in Georgian healthcare settings. Statistical tests comparing resistance rates among different antibiotic classes revealed significant differences (p<0.05) (Figure 3).

Among *Streptococcus pneumoniae* isolates, resistance to penicillin was universal, with all 45 isolates demonstrating resistance. Genetic analysis was performed using PCR, employing primers for pbp1a, pbp2x, and pbp2b adopted from previously published studies. Specifically, primers for pbp1a were sourced from Granger et al. (2006) [16], while primers for pbp2b and pbp2x were obtained from Ardanuy et al. (2014) [17]. Primers for tetM were designed based on sequences available in the literature [18]. Mutations in pbp1a, pbp2x, and pbp2b were identified in 93.3% (42/45) of the isolates. Resistance to macrolides was observed in 82.2% (37/45) of the isolates, mediated by the presence of the erm(B) gene. Similarly, 82.2% (37/45) of isolates exhibited resistance to tetracyclines due to the tetM gene. Resistance to fluoroquinolones was less prevalent, identified in 28.9% (13/45) of the isolates. Notably, one isolate of *Streptococcus pneumoniae* exhibited resistance to vancomycin, further highlighting the rise of MDR strains. Significant variations in resistance between antibiotic classes were observed (p<0.05) (Figure 3).

The study also assessed resistance patterns in gram-negative pathogens. Among the 203 *Pseudomonas aeruginosa* isolates, 79.8% (162/203) were ESBL-positive, with colistin resistance observed in 5.4% (11/203) of isolates. In *Klebsiella pneumoniae*, 65.1% (41/63) of isolates were ESBL-positive, while colistin resistance was noted in 3.2% (2/63). In *Acinetobacter baumannii*, 96.9% (63/65) of isolates demonstrated ESBL production, and 20% (13/65) exhibited resistance to colistin. Comparative analysis of ESBL and colistin resistance across the three pathogens revealed statistically significant differences (p<0.01).

These findings reveal that gram-positive bacteria, particularly *Staphylococcus aureus* and *Streptococcus pneumoniae*, contribute substantially to the burden of nosocomial pneumonia in Georgia, with concerning levels of resistance to key antibiotics. The presence of resistance genes, including mecA, pbp mutations, erm(B), and tetM, underscores the significant genetic adaptations that facilitate MDR phenotypes. Statistical analyses further highlight the importance of focusing on antibiotic stewardship and infection control measures in Georgian healthcare settings.

## Discussion

This study underscores the significant burden of nosocomial pneumonia caused by gram-positive pathogens in Georgian healthcare settings, with *Staphylococcus aureus* and *Streptococcus pneumoniae* emerging as critical contributors. The high prevalence of antimicrobial resistance observed in these pathogens reflects a growing global trend and highlights the urgent need for enhanced infection control practices and antimicrobial stewardship programs to mitigate the spread of MDR bacteria [9].

The prevalence of *Staphylococcus aureus* as the leading gram-positive pathogen (21.3%) aligns with previous studies that have highlighted its role as a major cause of hospital-acquired infections due to its versatility and ability to acquire resistance mechanisms [1,4,5]. The universal production of penicillinase and the high prevalence of methicillin resistance (86.4%) mediated by the mecA gene further reinforce its status as a formidable nosocomial pathogen [6,7]. Resistance to tetracyclines (90.3%) and fluoroquinolones (39.8%) was particularly notable in this study, limiting the options for empirical therapy. The detection of a vancomycin-resistant isolate is alarming, as vancomycin is often considered the last-resort antibiotic for MDR gram-positive infections [19]. These findings are consistent with global reports of rising vancomycin resistance in *Staphylococcus aureus* isolates, which poses significant challenges for clinicians [6,9,20].

For *Streptococcus pneumoniae*, universal resistance to penicillin mediated by mutations in pbp1a, pbp2x, and pbp2b genes aligns with findings from studies in Asia and Europe, where alterations in penicillin-binding proteins have been linked to reduced beta-lactam efficacy [7,10]. Resistance to macrolides (82.2%) and tetracyclines (82.2%) in *Streptococcus pneumoniae* isolates was similarly concerning, particularly given the widespread use of these antibiotics for respiratory infections. The presence of erm(B) and tetM genes underscores the role of horizontal gene transfer in spreading resistance among clinical isolates [15,21]. Fluoroquinolone resistance, though less prevalent (28.9%), represents a growing issue, as these antibiotics are often considered second-line treatments for pneumococcal infections [22,23].

Beyond gram-positive pathogens, this study highlights the concerning resistance profiles of gram-negative bacteria, including *Pseudomonas aeruginosa*, *Klebsiella pneumoniae*, and *Acinetobacter baumannii*. The high prevalence of ESBL-producing *Pseudomonas aeruginosa* (79.8%) and *Acinetobacter baumannii* (96.9%) reflects global trends, particularly in resource-limited settings where overuse of antibiotics often drives resistance [8,24]. Colistin resistance, observed in 5.4% of *Pseudomonas aeruginosa* isolates and 20% of *Acinetobacter baumannii* isolates, is particularly worrisome, as colistin is often considered a last-resort treatment for MDR gram-negative infections [12,13]. These findings align with previous studies in Europe and Asia that have reported similar rates of resistance and underscore the need for alternative treatment strategies [10,14].

The combination of MALDI-TOF MS and API tests for pathogen identification in this study provides a robust methodological framework, ensuring high accuracy and reproducibility. MALDI-TOF has been increasingly recognized as a gold standard for rapid pathogen identification, particularly in complex healthcare settings [11,20]. Meanwhile, API tests serve as a valuable adjunct for confirming resistance profiles in specific bacterial species, especially when MALDI-TOF alone may not suffice [12].

Adherence to the updated EUCAST 2024 guidelines in interpreting antimicrobial susceptibility ensures the clinical relevance of these findings. Standardizing laboratory practices according to international guidelines facilitates global comparability and improves the reliability of local surveillance data [13]. These insights can inform the development of region-specific treatment protocols, particularly in settings like Georgia, where antimicrobial resistance trends are not well-documented.

Clinical implications and future directions

The findings of this study emphasize the urgent need for comprehensive infection control measures, including active surveillance, antimicrobial stewardship programs, and strict adherence to hygiene protocols [25]. Targeted strategies to limit the spread of resistance genes, such as limiting the overuse of broad-spectrum antibiotics, should be prioritized. Furthermore, expanding molecular analyses to include whole-genome sequencing could provide deeper insights into the genetic mechanisms driving resistance in Georgian pathogens [26].

Limitations

This study has several limitations. The retrospective design may limit the ability to infer causation, and the exclusion of community-acquired pneumonia cases precludes comparisons with broader populations. Additionally, the focus on a limited set of resistance markers means that other potentially relevant genetic mechanisms may have been overlooked. Expanding future research to include larger sample sizes and additional resistance determinants is essential for a more comprehensive understanding of antimicrobial resistance trends.

## Conclusions

This study reveals alarming resistance patterns in gram-positive bacteria causing nosocomial pneumonia in Georgia, including universal penicillin resistance and high levels of resistance to macrolides and tetracyclines. The emergence of vancomycin-resistant isolates highlights the critical threat posed by MDR pathogens. These findings emphasize the need for urgent action, including enhanced infection control, surveillance, and targeted antibiotic stewardship. Advanced diagnostics like MALDI-TOF MS and adherence to EUCAST guidelines will aid in improving outcomes. Future research should focus on expanding molecular analyses and evaluating novel therapeutic approaches to combat antimicrobial resistance effectively.
